# Insulin resistance mediates the association between sleep duration and type 2 diabetes: Urban–rural evidence from a Chinese cohort study

**DOI:** 10.1097/MD.0000000000049092

**Published:** 2026-06-12

**Authors:** Zehua Wang, Aiping Zhao, Chenxing Ma, Zhen Zhang, Yazhong Zhang

**Affiliations:** a Endocrinology Dept. 2, Tangshan Central Hospital, Tangshan, Hebei, China.

**Keywords:** Chinese, cohort study, middle-aged and older adults, nighttime sleep duration, T2DM

## Abstract

This study aimed to examine urban–rural disparities in the association between nighttime sleep duration and type 2 diabetes mellitus (T2DM) risk in middle-aged and older adults, with particular attention to the mediating role of insulin resistance. We conducted a prospective cohort study using data from the China Health and Retirement Longitudinal Study, including 2169 participants. Nighttime sleep was categorized as short (<7 hours), normal (7–9 hours), or long (>9 hours). Urban/rural status was defined by self-reported residence. Cox proportional hazards models assessed associations between sleep duration and T2DM risk, and mediation analysis quantified indirect effects via insulin levels. Short sleep was significantly associated with increased T2DM risk in both urban (hazard ratio = 1.88, 95% confidence interval: 1.70–2.06) and rural (hazard ratio = 1.50, 95% confidence interval: 1.36–1.64) participants, compared with normal sleep. Baseline fasting insulin level (proxy for insulin resistance) mediated 20.3% of the total effect. The higher incidence observed in urban participants may be partly attributable to environmental stressors such as noise and air pollution. Short nighttime sleep was associated with a higher T2DM risk, with stronger effects among urban residents. Insulin resistance partially mediates this association. Population-specific interventions that promote sleep hygiene, address urban environmental stressors, and enhance rural healthcare access are warranted.

## 1. Introduction

Type 2 diabetes mellitus (T2DM) is a major global public health challenge, and China bears a particularly large and growing burden in the context of rapid demographic aging and lifestyle transitions. As of 2021, an estimated 140 million Chinese adults were living with diabetes, representing the largest national diabetes population worldwide.^[1,2]^ Middle-aged and older adults are disproportionately affected, consistent with age-related deterioration in glucose metabolism and insulin action.^[3,4]^ Given the substantial downstream impacts of T2DM, including cardiovascular morbidity, functional decline, and premature mortality, identifying modifiable risk factors that can be targeted in prevention strategies remains an urgent priority.^[1,5]^

In China, urban and rural disparities in socioeconomic conditions, health behaviors, and healthcare access are well documented and may contribute to heterogeneous diabetes risk.^[6]^ Urban residents often have better healthcare infrastructure and higher socioeconomic status, but may also experience greater exposure to sedentary behaviors, energy-dense diets, and psychosocial stressors that increase metabolic risk.^[7,8]^ Conversely, rural residents frequently face barriers to healthcare utilization and lower health literacy, which may delay recognition and management of metabolic disorders.^[9]^ Consistent with these structural differences, prior studies have reported higher diabetes prevalence in urban compared with rural settings, while rural regions tend to show higher proportions of undiagnosed or untreated cases.^[10,11]^ Understanding how modifiable lifestyle factors operate within these contrasting contexts is important for developing population-specific diabetes prevention strategies.

Sleep duration has emerged as a key behavioral determinant of cardiometabolic health, and both short and long nighttime sleep have been associated with increased T2DM risk through pathways involving hormonal dysregulation, circadian disruption, inflammation, and impaired insulin sensitivity.^[12–14]^ A growing body of evidence further indicates that sleep disturbance is closely tied to systemic inflammatory activity. For example, mediation-based analyses have linked sleep disturbance to inflammatory biomarker profiles within broader lifestyle pathways, such as sedentary behavior and exercise,^[15]^ and mechanistic syntheses highlight that disturbed sleep can activate inflammatory signaling and related downstream consequences.^[16]^ In parallel, T2DM is increasingly recognized as a chronic low-grade inflammatory state, with inflammatory markers and composite indices reflecting disease burden and complications.^[17,18]^ Importantly, inflammation is tightly intertwined with insulin resistance, as obesity-related inflammatory pathways and oxidative stress can impair insulin signaling and contribute to metabolic dysfunction.^[19,20]^ Together, these observations support a coherent biological framework in which altered sleep may promote inflammatory activation, exacerbate insulin resistance, and thereby increase the likelihood of developing T2DM.

Despite accumulating evidence linking sleep duration to diabetes, whether the association between sleep duration and T2DM differs by urban versus rural residence remains insufficiently understood, particularly among middle-aged and older adults.^[21]^ Urban populations may be more likely to experience sleep disruption due to environmental and psychosocial conditions, such as noise, light exposure, commuting demands, and stress, whereas rural populations may experience irregular sleep schedules shaped by occupational patterns and resource constraints.^[6,10,22]^ In addition, most prior studies are cross-sectional, limiting temporal inference, and few have examined potential mechanistic pathways, such as insulin-related mediation, within a longitudinal framework.^[23,24]^

To address these gaps, we used data from the China Health and Retirement Longitudinal Study (CHARLS), a nationally representative prospective cohort of Chinese adults aged 45 years and older, to investigate the association between nighttime sleep duration and incident T2DM, urban and rural disparities in this association, and the extent to which insulin-related pathways may mediate the observed relationship. By integrating stratified risk estimation with mediation analysis, this study aims to improve understanding of population-specific risk patterns and inform more targeted prevention strategies in both urban and rural settings.

## 2. Methodology

### 2.1. Data source

This study utilized data from the CHARLS, a nationwide survey conducted by the National Development Institute at Peking University. The survey aims to collect comprehensive health, socioeconomic, and demographic data from individuals aged 45 years and older. Initiated in 2011, CHARLS employed a multistage, stratified, and clustered sampling method to recruit participants from 28 provinces and 150 counties across China. Subsequent waves of data collection were conducted in 2013, 2015, 2018, and 2020, incorporating additional health assessments and self-reported health-related behaviors.

All participants provided written informed consent before participation, and the study was approved by the Biomedical Ethics Review Committee of Peking University (IRB00001052-11015).^[25]^

### 2.2. Study population

Participants were tracked across the 5 survey waves (2011–2020) using unique identifiers to consolidate their health and demographic data. The 2011 CHARLS baseline included 17,708 respondents. We first retained 13,790 participants who could be successfully linked to at least one follow-up wave (2013, 2015, 2018, or 2020) and had follow-up information on T2DM status; 3918 participants without linkable follow-up records or without follow-up T2DM information were excluded at this stage. For the primary association analyses, we excluded 790 participants with prevalent T2DM at baseline, 143 participants with missing nighttime sleep duration, and 0 participants with missing residence information. We then excluded 6262 participants with missing baseline covariate data in the 2011 survey, resulting in a primary analytic cohort of 6705 participants. For mediation analyses requiring baseline insulin, we further restricted the sample to participants with baseline insulin measurements available from the CHARLS biomarker subsample; 4536 of the 6705 participants lacked insulin data at baseline, leaving 2169 participants for mediation analyses.

**Figure 1.**
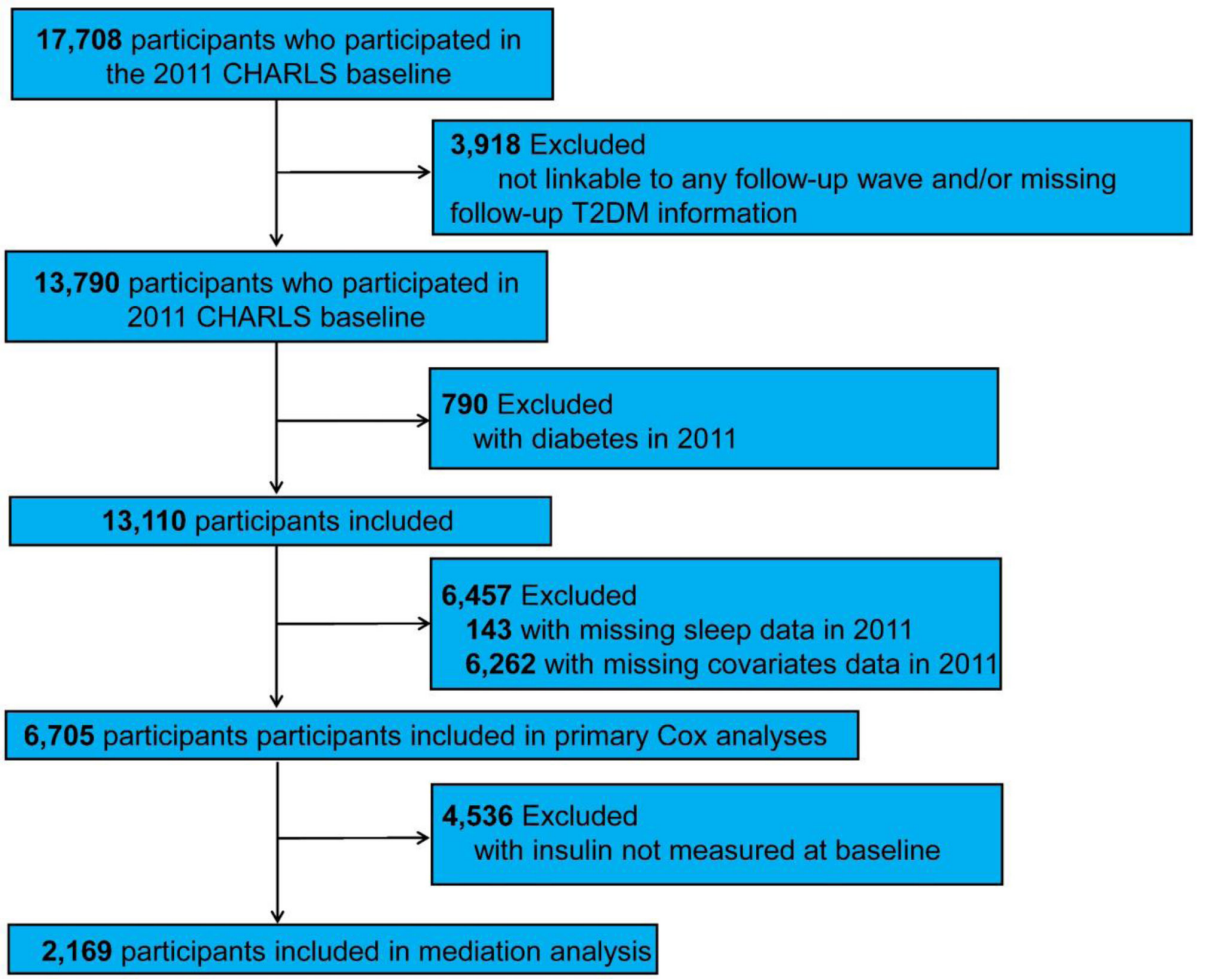
Flowchart of participant selection for the primary Cox analyses and the insulin mediation sub-cohort. CHARLS = China Health and Retirement Longitudinal Study.

The flow diagram illustrating participant selection is presented in Figure 1.

### 2.3. Exposure: urban–rural disparities

Urban–rural classification was based on participants’ self-reported residential location recorded in CHARLS at baseline. Participants were categorized into the following groups:

Urban group: individuals residing in county-level or higher administrative regions. Rural group: individuals residing in subcounty-level administrative regions.

This variable reflects an administrative and self-identified residential context rather than a direct measure of urbanicity (population density, environmental noise, air pollution, occupational structure, or community-level socioeconomic conditions). Given that urbanization in China is gradual and many communities are undergoing peri-urban development, some misclassification between urban and rural categories is possible, particularly for semi-urbanizing areas and individuals with residential mobility.

### 2.4. Nighttime sleep duration assessment

Nighttime sleep duration was self-reported at baseline using the standard CHARLS question: “During the past month, how many hours of actual sleep did you usually get at night (average hours for one night)?” Participants reported the average number of hours slept at night, which may be shorter than the time spent in bed. Based on established recommendations, nighttime sleep duration was categorized as short (<7 hours), normal (7–9 hours), or long (>9 hours). Because this measure was obtained using a single self-reported item, it may be subject to recall bias and reporting error. Objective sleep measurements such as actigraphy or polysomnography were not available in CHARLS, and detailed sleep phenotypes (sleep quality, insomnia symptoms, nocturnal awakenings, clinically assessed sleep apnea, and circadian timing) were not systematically captured; therefore, these factors could not be directly evaluated and may contribute to residual confounding. CHARLS also collected information on daytime napping using the question, “During the past month, how long did you take a nap after lunch (minutes)?” and we used this variable in sensitivity analyses to evaluate the robustness of the nighttime sleep findings.

### 2.5. T2DM and follow-up assessment

Incident T2DM was ascertained at each CHARLS wave using self-reported physician diagnosis. Participants were asked: “Has a doctor or physician ever told you that you have diabetes?” and “When was diabetes first diagnosed or known by yourself?” Participants were followed from the 2011 baseline survey to the first report of T2DM, death, or the last available follow-up in 2020. The first reported diagnosis date was treated as the event time, and participants without T2DM were censored at their last follow-up interview. Because standardized biochemical testing (fasting plasma glucose, glycated hemoglobin, or oral glucose tolerance test) and medical record verification were not available in a consistent and comparable manner for incident case ascertainment across all follow-up waves, we used the self-reported physician diagnosis measure to ensure harmonized outcome definition over time.

### 2.6. Covariates

The study adjusted for potential confounders measured at baseline, including demographic variables (age group, sex, education level, and marital status), lifestyle behaviors (smoking status and alcohol consumption), and socioeconomic and health-related variables (type of medical insurance, body mass index category, number of living children, income level, and employment status). In addition, we included baseline physical activity and psychosocial status to strengthen confounding control. Physical activity was assessed using CHARLS items on the frequency of vigorous activity, moderate activity, and walking, and was categorized into ordered levels of activity (e.g., none, occasional, and frequent) based on reported frequency. Depressive symptoms were assessed using the 10-item Center for Epidemiologic Studies Depression Scale (CES-D-10) and were modeled as a binary indicator using a commonly applied cutoff (CES-D-10 ≥ 10) or as an ordinal category depending on distribution. These variables were included in the fully adjusted model.

### 2.7. Statistical analysis

Baseline characteristics of participants were summarized as frequencies (n) and percentages (%). The cumulative incidence of T2DM across urban–rural groups and nighttime sleep duration categories was estimated using the Kaplan–Meier method. Cox proportional hazards (PH) regression models were employed to calculate hazard ratios (HRs) and 95% confidence intervals (CIs) for T2DM risk associated with urban–rural residence and sleep duration. The PH assumption was evaluated for the primary Cox models using Schoenfeld residual-based tests and was confirmed to be met the assumption (Global PH test *P* value = .189).

Three models were constructed to assess the interaction between urban–rural disparities and sleep duration:

Model I: No covariates adjusted.
Model II: Adjusted for age and sex.
Model III: Fully adjusted for all covariates.

The interaction between urban–rural residence and nighttime sleep duration was tested using the Wald test. A stratified analysis was performed with rural residents with normal sleep as the reference group. Results were visualized using forest plots.

Simultaneously, mediation analysis was conducted to evaluate whether baseline fasting insulin level, as a proxy of insulin resistance, partially mediated the association between nighttime sleep duration and incident T2DM. Venous blood collection in CHARLS was scheduled in the morning, and respondents were asked to fast overnight; fasting status at blood draw was recorded, and mediation analyses were restricted to participants with available baseline insulin measurements from the biomarker subsample and with fasting status recorded.^[26]^ Because insulin distributions are typically right-skewed, insulin was modeled on the log scale in mediation models. The indirect effect was estimated using the product-of-coefficients approach with bootstrap resampling (5000 iterations) to obtain CIs for the mediated proportion. We emphasize that causal interpretation of mediation estimates requires strong assumptions, including no unmeasured confounding of the exposure-mediator and mediator-outcome relationships and correct model specification; therefore, mediation results are presented as supportive evidence of a potential pathway rather than definitive causal decomposition. Since insulin was measured at baseline only, mediation reflects the contribution of baseline metabolic status and cannot capture time-varying changes in insulin resistance during follow-up.^[27]^

For sensitivity analysis, it should be noted that the primary analyses were conducted using a complete-case approach because the Cox models required a consistent set of covariates across participants. To evaluate potential selection bias due to missing baseline covariates, we additionally performed multiple imputation by chained equations under a missing-at-random assumption, generating 20 imputed datasets. The imputation model included nighttime sleep duration, residence, T2DM event indicator and follow-up time, insulin (where applicable), and all baseline covariates included in Model III. Cox models were fit within each imputed dataset and combined using Rubin rules. Another sensitivity analysis is we interpret urban–rural differences as broad contextual contrasts and used this classification to examine whether residential context modifies the association between nighttime sleep duration and T2DM risk. As an additional robustness check for potential urban–rural misclassification, we repeated the fully adjusted models using baseline hukou type (agricultural vs nonagricultural household registration) as an alternative proxy for rural/urban context. Simultaneously, we additionally adjusted for baseline post-lunch napping duration (minutes/day) to assess whether daytime napping influenced the association between nighttime sleep duration and incident T2DM. The primary conclusions were evaluated for consistency under this expanded adjustment set. In additional sensitivity analyses, we further adjusted for baseline healthcare utilization proxies available in CHARLS, including recent health examination and/or outpatient or inpatient service use to evaluate whether differential diagnostic opportunity influenced the urban–rural pattern of associations.

All analyses were performed in Stata 16.0 and R 4.3.2, with a *P*-value of < .05 considered statistically significant.

## 3. Results

### 3.1. Baseline characteristics

Of the 2169 participants included in this study, 1372 (63.3%) lived in rural areas and 797 (36.7%) resided in urban areas. The majority were younger than 70 years old (85.2%), and male participants accounted for 47.7% of the sample, slightly more common in rural areas. Rural participants had lower educational attainment, with 30.2% being illiterate and 46.4% having completed only primary school or below, while urban participants had higher levels of education, with 37.3% completing middle school and 7.4% attending technical school or above. Most participants were married (87.0%), and rural residents were more likely to still smoke and consume alcohol, whereas urban residents had a higher proportion of never-smokers. Rural participants were predominantly covered by New Cooperative Medical Insurance (90.2%) and more likely to report no income (72.5%), whereas urban participants had higher income levels (42.8%) and were more likely to be unemployed (45.8%) or engaged in nonagricultural jobs (25.3%). Nighttime sleep duration was slightly longer in urban areas, but short sleep duration (<7 hours) was more prevalent among rural residents. The detailed baseline characteristics are presented in Table 1.

**Table 1:** Basic characteristics of participants.

Variables	Overall sample (n = 2169)			
	Total (n, %)	Rural (n = 1372)	Urban (n = 797)	*P*-value
Age groups (yrs)				.116
<70	1847 (85.2)	1167 (85.1)	680 (85.3)	
70–80	271 (12.5)	166 (12.1)	105 (13.2)	
≥80	51 (2.4)	39 (2.8)	12 (1.5)	
Gender (male)	1035 (47.7)	679 (49.5)	356 (44.7)	.034
Educational level				<.001
Illiterate	553 (25.5)	415 (30.2)	138 (17.3)	
Primary school and below	939 (43.3)	636 (46.4)	303 (38.0)	
Middle school and high school	604 (27.8)	307 (22.4)	297 (37.3)	
Technical school and above	73 (3.4)	14 (1.0)	59 (7.4)	
Marriage (married)	1888 (87.0)	1195 (87.1)	693 (87.0)	.974
Smoke				.015
Never smoking	1333 (61.5)	817 (59.5)	516 (64.7)	
Former smoking	175 (8.1)	107 (7.8)	68 (8.5)	
Still smoking	661 (30.5)	448 (32.7)	213 (26.7)	
Drink alcohol (current)	757 (34.9)	497 (36.2)	260 (32.6)	.099
Medical insurance				<.001
UEMI*	140 (6.5)	71 (5.2)	69 (8.7)	
URRMI*	174 (8.0)	23 (1.7)	151 (18.9)	
URMI*	31 (1.4)	17 (1.2)	14 (1.8)	
GMI*	74 (3.4)	8 (0.6)	66 (8.3)	
NCMI*	1695 (78.1)	1238 (90.2)	457 (57.3)	
Other medical insurance	55 (2.5)	15 (1.1)	40 (5.0)	
Income group				<.001
Non-income*	1390 (64.1)	995 (72.5)	395 (49.6)	
Low income*	86 (4.0)	65 (4.7)	21 (2.6)	
Middle income*	131 (6.0)	91 (6.6)	40 (5.0)	
High income*	562 (25.9)	221 (16.1)	341 (42.8)	
Employment status				<.001
Unemployed	738 (34.0)	373 (27.2)	365 (45.8)	
Agricultural job	1152 (53.1)	922 (67.2)	230 (28.9)	
Nonagricultural job	279 (12.9)	77 (5.6)	202 (25.3)	
Physical activity level				<.001
Low	525 (24.2)	206 (15.0)	319 (40.0)	
Moderate	691 (31.9)	412 (30.0)	279 (35.0)	
High	953 (43.9)	754 (55.0)	199 (25.0)	
Depressive symptoms				<.001
No	1482 (68.3)	892 (65.0)	590 (74.0)	
Yes	687 (31.7)	480 (35.0)	207 (26.0)	
Sleep duration (hr)	5.04 (1.15)	4.99 (1.19)	5.14 (1.08)	.002
Sleep duration group (short)	1447 (66.7%)	1011 (73.7%)	436 (54.7%)	<.001
Insulin level (pmol/L)	81.05 (44.77)	85.03 (56.32)	76.80 (33.28)	<.001

Values are presented as n (%) for categorical variables and mean (SD) for continuous variables. *P*-values compare rural and urban participants. Non-income indicates no personal annual income; low income, personal annual income of 0 to 20,000 CNY; middle income, 20,000 to 50,000 CNY; high income, >50,000 CNY. Short sleep duration was defined as <7 hours/night.

GMI = Government Medical Insurance, NCMI = New Cooperative Medical Insurance, UEMI = Urban Employee Medical Insurance, URMI = Urban Resident Medical Insurance, URRMI = Urban and Rural Resident Medical Insurance.

* Indicates a category/level of the corresponding variable and does not denote statistical significance.

### 3.2. Nighttime sleep duration with the risk of T2DM grouped by urban/rural residence

**Figure 2.**
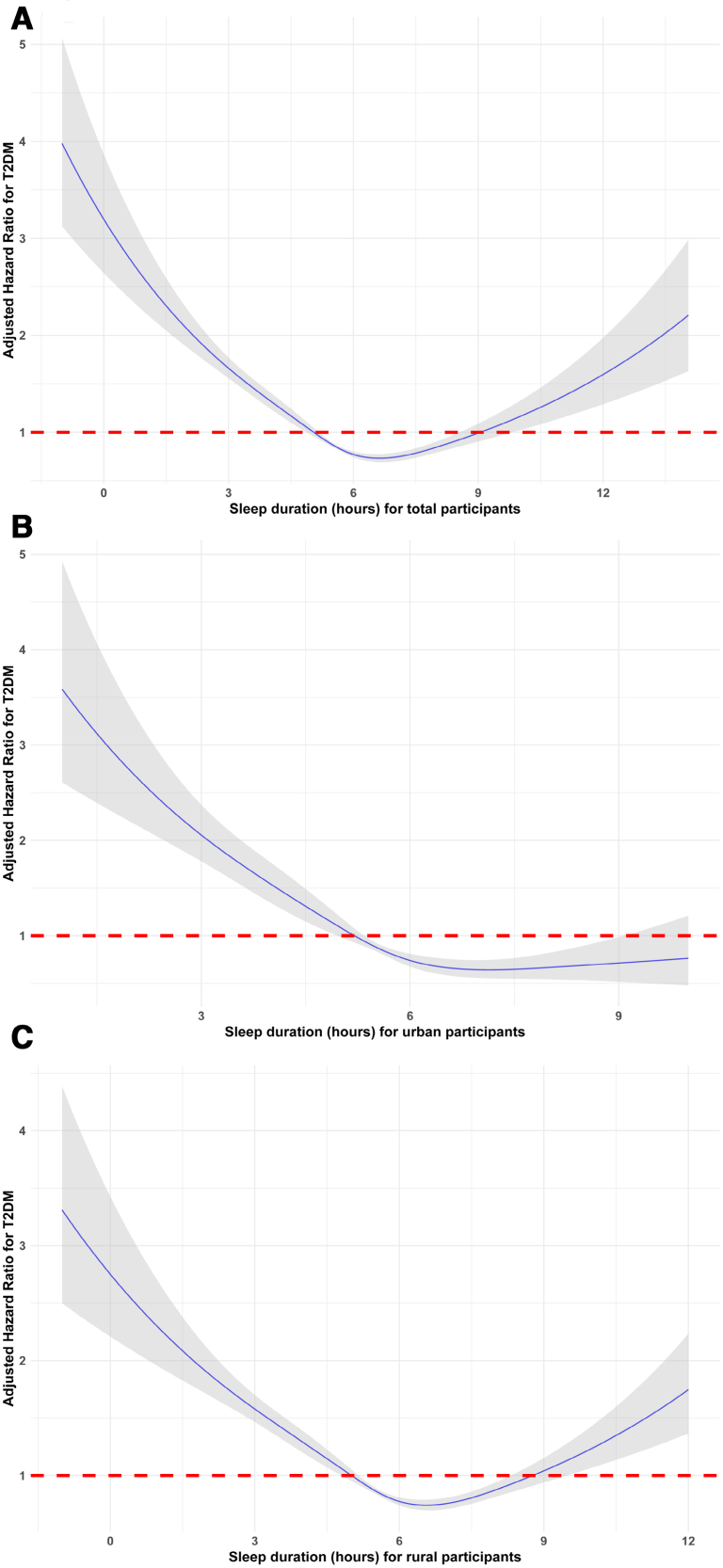
Dose–response relationship between nighttime sleep duration and T2DM among Chinese middle-aged and older adults grouped by urban/rural residence. (A) Total participants. (B) Urban participants. (C) Rural participants. Adjusted for age group, gender, educational level, marital status, medical insurance, smoking, drinking alcohol, income group, employment status. T2DM = type 2 diabetes mellitus.

A total of 454 participants developed T2DM during the follow-up period. Table 2 presents the association between nighttime sleep duration and the risk of T2DM stratified by urban and rural residence. In all 3 models, short sleep duration (<7 hours) was associated with an increased risk of T2DM compared to the reference group (7–9 hours, enough sleep) for both rural and urban participants. Simultaneously, among rural residents, short sleepers exhibited a 62%, 58%, and 50% increased risk of developing T2DM in Models I, II, and III, respectively (Model III: HR = 1.50, 95% CI: 1.36–1.64). Similarly, among urban residents, short sleepers demonstrated an even higher increased risk, with HRs of 2.02, 1.97, and 1.88 in Models I, II, and III, respectively (Model III: HR = 1.88, 95% CI: 1.70–2.06). The incidence rate of T2DM was higher in urban short sleepers (32.89 per 1000 PYs) compared to rural short sleepers (29.45 per 1000 PYs). Notably, the cumulative incidence of T2DM was also higher for urban residents with short sleep compared to their rural counterparts. Figure 2 shows the dose–response relationship between nighttime sleep duration and T2DM among Chinese middle-aged and older adults grouped by urban/rural residence.

**Table 2:** Associations between study participants in different nighttime sleep duration subgroups and T2DM. Values are hazard ratios (95% confidence intervals) unless otherwise stated.

Variables	Sleep duration	Participants (N)	Events (n)	Incidence rate (per 1000 PYs)	Model I HR (95% CI)	Model II HR (95%CI)	Model III HR (95% CI)
Rural							
	Enough sleeper (≥ 7h)	361	62	18.2	1.00	1.00	1.00
	Short sleeper (<7h)	1011	221	29.45	1.62 (1.38–1.90)	1.58 (1.34–1.87)	1.50 (1.36–1.64)
Urban							
	Enough sleeper (≥7h)	361	66	20.18	1.00	1.00	1.00
	Short sleeper (< 7h)	436	105	32.89	2.02 (1.45–2.59)	1.97 (1.40–2.54)	1.88 (1.70–2.06)

Short sleeper was defined as nighttime sleep duration <7 hours; enough sleeper was defined as ≥7 hours and included participants with normal sleep (7–9 hours) and long sleep (>9 hours), which were not modeled separately. In the stratified analyses, enough sleepers within each residence stratum served as the reference group. Model I, no covariates were adjusted; Model II, adjusted for age group and gender; Model III, adjusted for age group, gender, educational level, marital status, medical insurance, smoking, drinking alcohol, number of living children, income, and employment status; residence was not included because models were stratified by residence.

CI = confidence interval, HR = hazard ratio, PYs = person-years, T2DM = type 2 diabetes mellitus.

### 3.3. Mediation analysis

**Figure 3.**
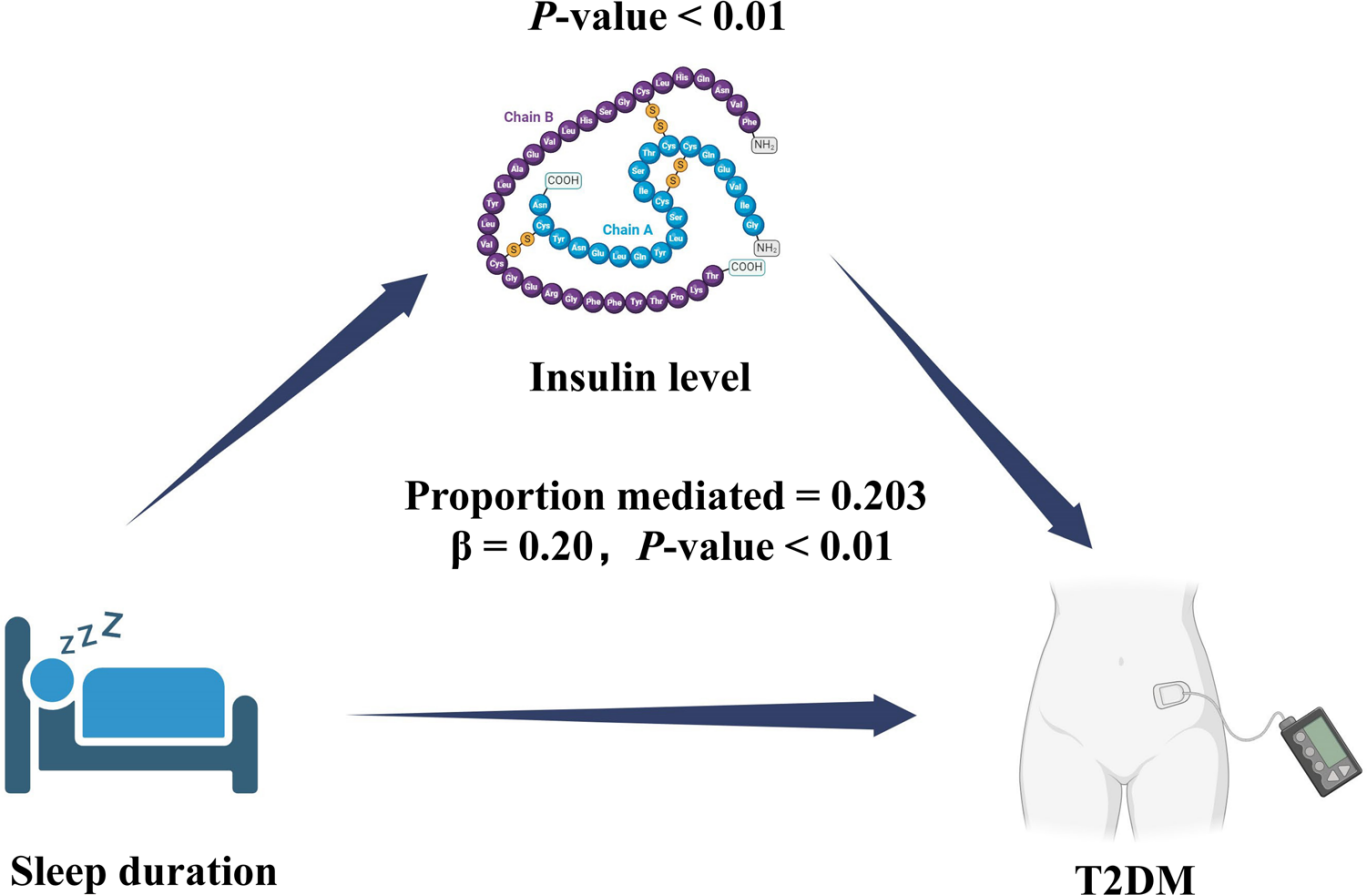
Path diagram of mediation analysis of relationship between sleep duration, insulin level and T2DM. T2DM = type 2 diabetes mellitus.

The mediation analysis indicated that baseline fasting insulin level, used as a proxy for insulin resistance, significantly mediated the association between nighttime sleep duration and incident T2DM (Fig. 3). Specifically, the indirect pathway through baseline insulin accounted for 20.3% of the total effect of nighttime sleep duration on T2DM risk (indirect effect: β = 0.20, *P* < .01), suggesting partial mediation. These findings should be interpreted as reflecting mediation via baseline metabolic status because insulin was measured only at baseline and may vary over the follow-up period. In contrast, insulin did not significantly mediate the associations between other factors, such as urban versus rural residence, and T2DM risk (all *P*-values > .05).

We also reestimated the fully adjusted sleep–T2DM models within the insulin-measured biomarker subcohort, and the direction and magnitude of associations were consistent with those observed in the primary analytic cohort. To further assess potential selection into the biomarker subsample, we conducted an inverse probability weighted analysis based on observed baseline characteristics predicting insulin measurement availability, and the mediation and main-effect inferences were materially unchanged.

### 3.4. Sensitivity analysis

A series of sensitivity analyses were conducted to assess the robustness of the primary findings. First, we compared complete-case estimates with results from multiple imputation for missing baseline covariates (and sleep duration where applicable); pooled associations were materially unchanged. For example, in the fully adjusted model, the HR for short versus normal sleep remained elevated in both rural and urban participants after imputation (rural: HR = 1.47, 95% CI: 1.34–1.61; urban: HR = 1.85, 95% CI: 1.68–2.03), closely aligning with the complete-case estimates (rural: HR = 1.50, 95% CI: 1.36–1.64; urban: HR = 1.88, 95% CI: 1.70–2.06). We also examined missingness patterns by residence and compared baseline characteristics between included and excluded participants; although excluded participants tended to have lower socioeconomic indicators, the direction and inference of the sleep–T2DM association were consistent in the imputed analyses, suggesting that complete-case selection was unlikely to fully account for the observed associations. Second, to evaluate the potential influence of urban–rural misclassification, we reestimated the fully adjusted models using the baseline hukou type (agricultural vs nonagricultural household registration), and the direction and magnitude of the associations remained similar (rural: HR = 1.46, 95% CI: 1.33–1.60; urban: HR = 1.83, 95% CI: 1.66–2.01). Third, we additionally adjusted for baseline post-lunch napping duration (minutes/day) to assess whether daytime napping influenced the association between nighttime sleep duration and incident T2DM, and the effect estimates remained materially unchanged (*P* > .05). Fourth, competing-risk approaches that estimate cumulative incidence (Fine-Gray model) were considered; however, mortality ascertainment and death timing in CHARLS during the study period are not sufficiently complete or precise for valid cumulative-incidence modeling in our analytic dataset (publicly available exit/end-of-life information is not consistently available across waves, and exact death dates are often missing or interval-known).^[28,29]^ Under these constraints, competing-risk regression would require strong assumptions or model-dependent imputation of death times, which could introduce additional bias. We therefore focused on cause-specific hazards of incident T2DM among those still at risk and under observation, censoring participants at their last confirmed interview. Finally, after additionally adjusting for baseline healthcare utilization proxies, the associations between short nighttime sleep and incident T2DM and the urban–rural pattern of effect estimates remained materially unchanged, suggesting that differential diagnostic opportunity was unlikely to fully explain the observed findings. Overall, these sensitivity analyses indicated that the estimated association between nighttime sleep duration and incident T2DM was robust to different missing-data handling strategies and alternative urbanicity definitions, with no meaningful change in inference (all comparisons yielded nonsignificant differences in effect estimates, *P* > .05).

## 4. Discussion

This study investigated the urban–rural disparities in the relationship between nighttime sleep duration and the risk of T2DM among middle-aged and older adults in China. Our findings indicated that both urban and rural residents with short sleep duration were at a significantly higher T2DM odds compared to those with normal sleep duration. Notably, urban residents exhibited a greater HR, reflecting the compounded influence of urban-specific stressors.

The elevated T2DM odds observed among short sleepers align with existing literature indicating that insufficient sleep adversely affects glucose metabolism. However, the urban–rural differences identified in our analysis warrant further discussion. Urban residents demonstrated a higher HR for T2DM associated with short sleep compared to rural residents (HR: 1.88 vs 1.50 in fully adjusted models). This disparity could be attributed to the cumulative effects of urban stressors, including environmental noise, air pollution, and occupational demands, which are known to exacerbate metabolic dysregulation.^[30–32]^ On the other hand, rural residents faced challenges such as lower health literacy and limited access to healthcare services, potentially leading to delayed diagnoses and poorer management of T2DM risk factors.^[9]^ These structural inequities underline the importance of targeted interventions tailored to the specific needs of urban and rural populations.

The mediation analysis demonstrated that insulin levels accounted for 20.3% of the effect of sleep duration on T2DM risk, consistent with the well-documented role of insulin resistance in the pathogenesis of T2DM.^[33,34]^ Insufficient sleep disrupts circadian rhythms, impairing β-cell function and reducing glucose tolerance, which are critical for maintaining glucose homeostasis.^[35]^

The biological mechanisms linking short sleep duration to T2DM involve several interrelated pathways. Firstly, insufficient sleep disrupts the hypothalamic–pituitary–adrenal axis, leading to chronic hypercortisolemia, which promotes insulin resistance and adiposity. Elevated cortisol levels impair insulin signaling by reducing glucose uptake in muscle cells and enhancing hepatic gluconeogenesis.^[36,37]^ Secondly, sleep deprivation alters the secretion of appetite-regulating hormones, such as reduced leptin (satiety hormone) and increased ghrelin (hunger hormone), promoting overeating and subsequent weight gain, both of which are key risk factors for T2DM.^[38,39]^ Additionally, short sleep duration is associated with increased levels of pro-inflammatory cytokines such as interleukin-6 and tumor necrosis factor-alpha, which exacerbate insulin resistance and pancreatic β-cell dysfunction.^[40]^. These mechanisms highlight how metabolic and inflammatory dysregulation induced by inadequate sleep contributes to the pathophysiology of T2DM. Beyond these pathways, short sleep can also impair circadian regulation of glucose metabolism, reducing insulin sensitivity in peripheral tissues and altering the timing of hormonal secretion that supports glycemic control, thereby increasing postprandial glucose excursions. In parallel, sleep restriction has been linked to heightened sympathetic nervous system activity and reduced nocturnal blood pressure dipping, which may further aggravate metabolic stress and vascular dysfunction that commonly co-occur with T2DM. Collectively, these multifactorial mechanisms provide biological plausibility for the observed association between short nighttime sleep and elevated T2DM risk, and they also support the interpretation that insulin-related pathways may partially explain the sleep-T2DM relationship in middle-aged and older adults.^[41–43]^

This study’s strengths include its use of a nationally representative cohort and the adjustment for comprehensive confounders and a series of sensitivity analyses, enhancing the generalizability and robustness of the findings. However, the reliance on self-reported sleep data introduces potential recall bias. Our urban–rural classification was based on self-reported administrative residence and does not directly measure urbanicity-related exposures such as population density, noise, air pollution, occupational structure, or community socioeconomic conditions; peri-urbanization and residential mobility may therefore introduce misclassification, which could attenuate or obscure true contextual heterogeneity in the sleep–T2DM association. Future studies should incorporate objective measures such as polysomnography or actigraphy for more precise sleep assessments. Additionally, the observational design precludes definitive causal inferences. Longitudinal interventional studies are required to establish causality. Simultaneously, nighttime sleep duration was self-reported using a single questionnaire item and we lacked objective sleep measures and detailed information on sleep quality and sleep disorders, which may introduce measurement error and residual confounding; although adjustment for post-lunch napping in sensitivity analyses did not materially change the results, unmeasured sleep phenotypes remain an important limitation.^[44]^ Furthermore, residual confounding is possible because detailed diet/carbohydrate intake, shift-work history, and sleep medication or sedative use were not consistently available in a harmonized manner for this cohort and therefore could not be adjusted.^[45]^ Correspondingly, because objective environmental exposures (such as air pollution, noise, and light-at-night)^[46]^ were not measured and could not be reliably linked at high spatial resolution in the public-use CHARLS data, we could not directly test environmental mechanisms underlying the urban–rural differences observed in this study. Finally, T2DM was identified using self-reported physician diagnosis rather than repeated standardized biochemical measurements or medical record verification; therefore, undiagnosed cases may have been missed, and differential healthcare access between urban and rural residents could introduce detection bias that may partially contribute to urban–rural differences in observed incidence.

Our findings highlight the urgent need for targeted interventions to mitigate urban-specific stressors and improve rural healthcare access. Policymakers should prioritize strategies that promote sleep hygiene, reduce environmental stressors, and facilitate early diabetes screening, particularly in underserved rural communities. Further research is also needed to elucidate the molecular mechanisms linking sleep duration to metabolic health.^[47,48]^

## 5. Conclusion

This study reveals a significant association between short nighttime sleep duration and the higher odds of T2DM, with urban residents showed a stronger association between short nighttime sleep and T2DM risk; however, this contrast may reflect both contextual risk differences and differential diagnosis related to healthcare access. Insulin resistance partially mediates this relationship, highlighting its role in the sleep-T2DM pathway. Tailored interventions to improve sleep hygiene, reduce urban stressors, and enhance rural healthcare access are crucial for mitigating diabetes risk. Future research should focus on elucidating molecular mechanisms and assessing the impact of sleep-focused strategies on T2DM prevention.

## Acknowledgments

The views expressed in this publication are those of the authors.

## Author contributions

**Data curation:** Zehua Wang.

**Methodology:** Aiping Zhao.

**Software:** Chenxing Ma.

**Writing – original draft:** Zhen Zhang, Yazhong Zhang.

**Writing – review & editing:** Zhen Zhang, Yazhong Zhang.
